# The Feasibility of Using Virtual Reality and Eye Tracking in Research With Older Adults With and Without Alzheimer's Disease

**DOI:** 10.3389/fnagi.2021.607219

**Published:** 2021-06-28

**Authors:** Rebecca Davis

**Affiliations:** Kirkhof College of Nursing, Grand Valley State University, Grand Rapids, MI, United States

**Keywords:** Alzheimer's disease, aging, spatial cognition, virtual navigation, eye tracking, feasibility

## Abstract

**Aim:** To examine the feasibility of using large scale spatial, self-mobile, virtual reality, and eye tracking in older adults with and without Alzheimer's disease (AD).

**Methods:** Older adults with early stage AD (*n* = 38) and a control group without AD (*n* = 50) were asked to find their way in a large, projected VR simulation of a retirement community repeatedly over 10 trials for each of 2 days, while wearing eye tracking glasses. Feasibility measures, including tolerance, side effects, and ability to complete the VR and eye tracking were collected. This study reports the analysis of the feasibility data for the VR and eye tracking and comparison of findings between the groups.

**Results:** Over 80% of the subjects were able to complete the VR portion of the study. Only four subjects, all in the AD group, could not use the joystick and were excluded. Withdrawal rate (18%) was similar between the groups [*X*^2^_(2)_ = 2.82, *N* = 88, *p* = 0.245] with most withdrawals occurring after the fourth trial. Simulation sickness was not significantly different between the groups. Only 60% of the subjects had completed eye tracking videos; more subjects in the AD group had complete eye tracking videos than the control group; *X*^2^_(1)_ = 7.411, *N* = 88, *p* = 0.006. Eye tracking incompletion was primarily due to inability to calibration issues.

**Conclusion:** Virtual reality testing and eye tracking can be used in older adults with and without AD in a large-scale way-finding task, but that there are some limitations.

## Introduction

A major symptom of Alzheimer's disease (AD) is that individuals often become lost in both familiar or unfamiliar places, a symptom known as spatial disorientation (Caspi, 2014; Allison et al., 2016; Boccia et al., 2019). Spatial disorientation is likely due to the detrimental effect of AD on brain areas such as the hippocampus and related structures, which are necessary for finding one's way in an environment, an ability known as wayfinding (Allison et al., 2016). Wayfinding is an essential ability that is necessary for an individual to maintain independence in the world. Studies have shown that spatial disorientation is an early, pre-symptomatic finding predictive of an individual developing AD (Konishi et al., 2018; Parizkova et al., 2018). Wayfinding has been shown to be affected by both the cognitive and physical abilities of the user, as well as qualities of the environment (Marquardt, 2011; Davis and Weisbeck, 2016). Thus, the development and testing of methods to measure wayfinding ability in persons with AD is essential in order to identify spatial disorientation and to develop interventions to promote effective wayfinding in this population.

### Virtual Reality

Testing wayfinding abilities in persons with AD has evolved from primarily pen and paper types of spatial tests to more ecologically appropriate virtual reality (VR) wayfinding tests. VR tests have been shown to be more predictive of clinical progression to AD than are other “pen and paper” cognitive measures (Bailey et al., 2018; Levine et al., 2020). VR tests are exhibited as digitally displayed, three-dimensional, experiential environments (Diersch and Wolbers, 2019). VR environments can be displayed in many ways, including head mounted devices, flat computer screens, and large projected screens (Sherman and Craig, 2003). Movement in these environments may take place automatically (viewing only) or by the participant using a joystick, walking on a treadmill, or using other devices such as a steering wheel and accelerator (Vlcek and Laczo, 2014; Diersch and Wolbers, 2019). Many wayfinding tests in VR are specifically designed to be sensitive to hippocampal function, thus requiring subjects to find a hidden platform (virtual water maze) or location, using external environmental cues (Moffat and Resnick, 2002; Morganti et al., 2008, 2013; Davis et al., 2017). Many studies have shown that VR can be used to test spatial navigation, especially using specific maze tasks, in older adults with and without dementia (Moffat and Resnick, 2002; Shukitt-Hale et al., 2004; Vembar et al., 2004; Mueller et al., 2008; Jheng and Pai, 2009; Schoenfeld et al., 2010; Tangen et al., 2015; Parizkova et al., 2018). Using VR for spatial navigation testing has led to a rich understanding of strategies and abilities in a wide range of age groups, among individuals with varying types of abilities (Diersch and Wolbers, 2019).

Underreported in the literature is the feasibility of VR testing on persons with AD. Although much VR research has been done in older persons and some with AD, the majority studies in persons with AD involve mobility in a small maze-like environment on a computer screen whereby all subjects are exposed to the same environment (i.e., Davis et al., 2017; Parizkova et al., 2018). Other studies have examined the acceptability of VR as a method of rehabilitation or enjoyment for persons with AD (Saredakis et al., 2020). However, few studies have examined the use of VR to assess wayfinding in more naturalistic, real world settings; which is important if VR can be used to provide information about the effects of AD on specific real-world functions.

The use of VR for testing can be difficult for the aging population, especially those with AD. The lack of experience with technology (Barnard et al., 2013), the potential for side effects such as simulation sickness (Arns and Cerney, 2005), the ability to use a computer joystick or other mobility device, and enjoyment or tolerance of the technology can affect the ability for older adults to successfully use VR in an experiment (Diersch and Wolbers, 2019).

### Eye Tracking

Another technology often used in concert with VR is eye tracking. Eye tracking allows investigators to analyze eye movements, such as saccades and fixations, in order to determine gaze patterns and visual attention to visual objects and scenes (Duchowski, 2002). Eye tracking has been used to determine spatial strategies in young persons and to measure eye movements in persons with various neurocognitive disorders (Andersen et al., 2012; Anderson and MacAskill, 2013). Eye tracking during virtual navigation has shown promising results but has primarily been limited to static types of spatial tests (reading or finding a static object on a computer screen) vs. during active wayfinding in large-scale space (Anderson and MacAskill, 2013; Seligman and Giovannetti, 2015). Large scale spatial environments can be difficult for eye tracking analysis because each individual's experience differs based on his or her navigation choices (Lappi, 2015); yet large scale spatial environments are more naturalistic and akin to the real world. In large scale space, similar to many computer games, there may be many choices that a subject can make. The users' experiences differ based on what they look at and where they go. The eye tracking data must be coded from the numerous routes, resulting in an expansive number of variables that must be analyzed to have meaning.

Changes in the aging eye may also contribute to eye tracking difficulty in older persons. Cataracts, prior eye surgeries, and multi-focal glasses may make the procedure challenging, especially when eye tracking glasses are used (Shamim et al., 2019). Persons with AD may not understand they cannot touch or move the eye tracking glasses during testing. These issues could impact the quality of the data as well as attrition of participants in the study (Nyström et al., 2013).

### Study Purpose

Recently, this research team conducted a study of wayfinding in a VR environment with eye tracking in older adults with and without AD (Davis et al., 2017; Davis and Sikorskii, 2020). Prior to conducting the study, there was very little in the literature about the feasibility of using VR environments that allowed for self-motion in persons with AD, and very little about feasibility of eye tracking in persons with AD. Information about feasibility of these measures in persons with AD is important so that the researcher can select the appropriate method of VR and eye tracking; select an appropriate sample size (anticipating potential withdrawals or other problems); and minimize side effects and other difficulties that may occur. Thus, the aim of this study was to examine the feasibility of using VR and eye tracking in older adults with early stage AD. In addition, the research team sought to determine if there were differences between the AD and control groups with respect to the feasibility measures.

## Materials and Methods

### Subjects

Subjects were a convenience sample of older adults aged 62 years or older, who had either no cognitive disease (control group); or early stage AD or mild cognitive impairment due to AD (AD group) diagnosed by established criteria (Albert et al., 2011; McKhann et al., 2011). Participants had to demonstrate visual acuity of 20/40 or better (indicating good vision) and a lack of color blindness. A total of 50 persons were recruited to participate in the control group, and 38 for the AD group (Davis et al., 2017). The study was approved by the University and hospital's Internal Research Review Boards.

### Procedure

A detailed description of the methods for the parent study has been previously reported (Davis et al., 2017; Davis and Sikorskii, 2020) and is discussed briefly here. After recruitment from the community, participants gave informed consent if they were able; for those who lacked consent capacity, assent was obtained along with informed consent by their decision makers. Subjects completed the Montreal Cognitive Assessment (MoCA) and a demographics survey. The participants were taught how to use a joystick, and given as much time as they wished to practice moving to a visual target on the screen. The research assistants provided instruction and explanation about how to move a joystick purposefully in a desired direction. Subjects practiced moving the joystick until they became comfortable with it, moving from one part of the practice VR environment to another under the supervision of the research assistant. In order to ascertain that subjects understood how to use the joystick and how to follow the task directions, the subjects were required to virtually “move” to a visual target on the screen, using the joystick, within 30 s prior to the start of the study. If they were unable to do so, they were given more practice time if they wished; and asked again. After two failures, the research team determined that individual would not to be able to participate in the study.

**Figure 1:**
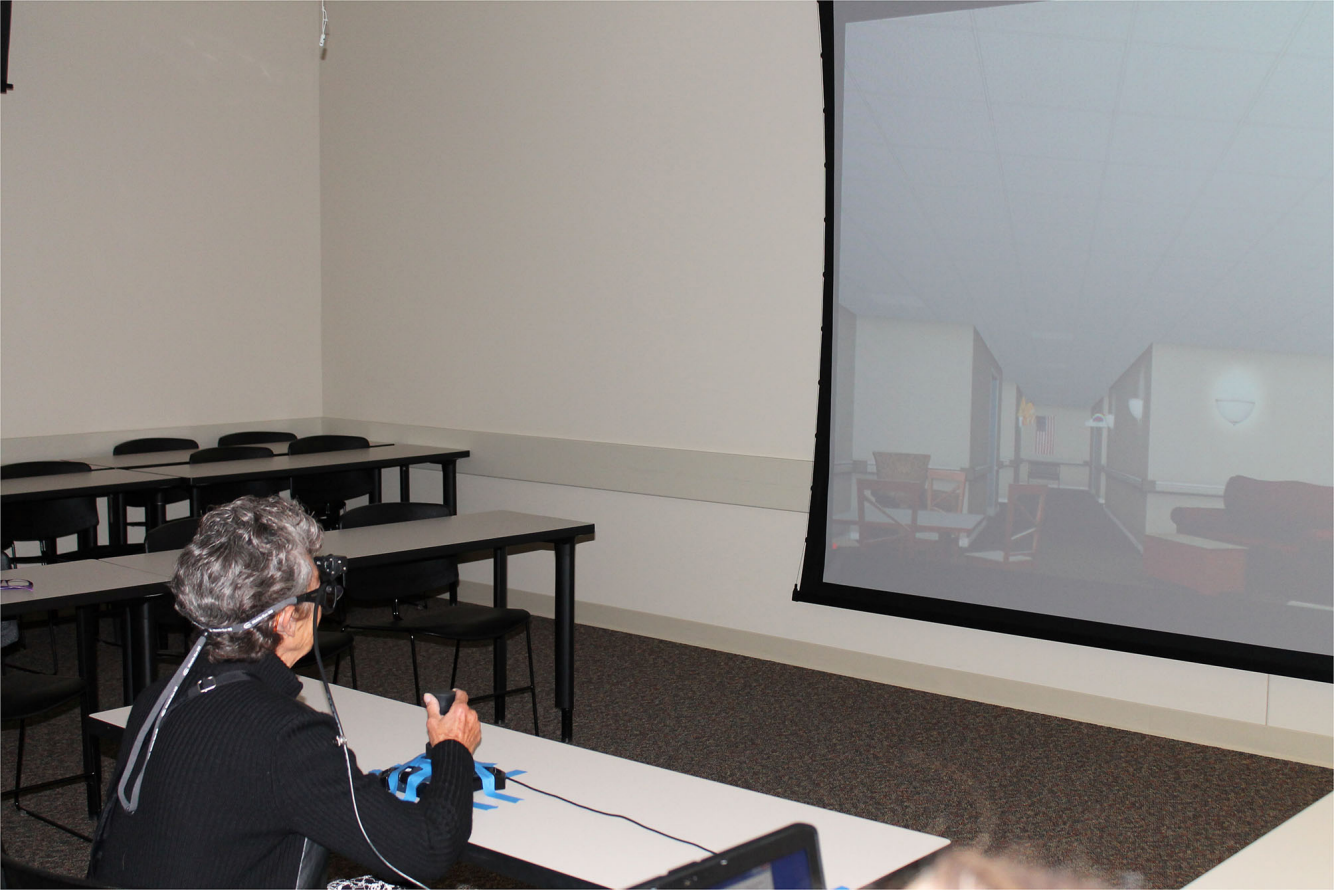
Projected virtual reality spatial navigation test with eye tracking.

Once subjects demonstrated an understanding of how to purposefully move within the VR environment with the joystick, they were asked to find their way to destinations repeatedly within two virtual environments while wearing eye tracking glasses, with the purpose of examining visual fixations while wayfinding. The VR environments, projected on a 12-foot screen, were realistic simulations of portions of a long-term care facility. The environments were equal in terms of distance and turns; but one environment contained visual cues specifically placed to assist with navigation and the other environment did not. Subjects used the joystick to move virtually while making choices at intersections to find the goal destinations (Figure 1). They could make wrong or right turns and were not corrected if they made a wrong turn, but they were shown the correct route from start to finish at the end of each trial to facilitate learning. Subjects were given a total of ten trials (5 trials in each environment) for each of two consecutive days. The trials ended at 3 min, or whenever the goal was found. Rest periods were given for 1 min between trials and 5 min between the two virtual environments (Davis et al., 2017).

For eye tracking, the researchers used an Applied Science Laboratory (ASL) eye tracking system (Mobile Eye) which tracked subjects' visual gaze during the wayfinding task (Applied Science Laboratories, 2015). The participants wore eye tracking glasses or goggles that went over the frame of their regular glasses. The eye tracking device had a camera mounted on the frame that recorded the VR environment in front of the participant; and another camera that recorded what the participant was focusing on by measuring pupil-corneal reflection via an optical device.

The eye tracking device was calibrated to each participant prior to use. This involved asking the participant to look at a standard set of images on a large display and accepting the fixation points once the eye was stable in the video feed with an acceptable reading of pupil tracking as detected and analyzed by the eye tracker computer and software (Nyström et al., 2013). The Mobile Eye has a calibration accuracy reported at 0.5 degrees (Applied Science Laboratories, 2015). Each participant that completed all of the trials had a total of 20 eye tracking videos (10 in each VR environment) over the two-day study. Recordings lasted the trial length of 3 min, or were stopped when the participant found the destination (Davis and Sikorskii, 2020).

Trained data collectors wrote notes during the VR/eye tracking portion of the study, indicating any problems that the subject had with the technology. Subjects were asked “how are you doing” after each 3-min trial and observed for any outward expressions of discomfort. If subjects said they were doing well, the data session continued. Those participants who reported feeling sick or having any problems were assessed; those who had simulation sickness symptoms or who were exhibiting or verbalizing any excessive frustration or other concerning issues were withdrawn from the study and the reasons why noted in the log. Therefore, subjects who had any complaints of simulation sickness were withdrawn from the study immediately. The reason for this is because of the vulnerable population included in the study (we did not want subjects to fall, or have any injuries due to vestibular symptoms that might be worsened if the study were to continue after symptoms were exhibited). Checklists were used to document any reasons for withdrawal from the study. After data were collected, the research team reviewed the videos of the VR session and eye tracking to determine complete vs. incomplete data and reasons documented for incomplete data.

### Feasibility Measures

Feasibility measures for the VR included: (1) the ability of the subjects to use a joystick (yes/no); (2) the number of withdrawals and why; and (3) the tolerance (side effects) of the VR as reported by the participants. Tolerance of the VR was measured by self-report after each of the 20 trials. For eye tracking, the feasibility measures included (1) the ability to calibrate the eye tracker as documented by the data collector; (2) participants' ability to refrain from adjusting/moving the glasses after calibration as indicated by the loss of eye tracking on the eye tracking video and subsequent notations made by the data collector that the subject moved his or her glasses; and (3) to provide quality data (eye tracking video not usable/complete; partially usable/complete; or complete and usable).

### Demographics and Cognitive Measures

Participants completed a demographic survey with the variables of age, sex, and years of education. Subjects completed the Montreal Cognitive Assessment, which is a 30 item cognitive screening tool that is sensitive to mild cognitive impairment and AD (Nasreddine et al., 2005) as well as the Digit Span Forwad and Digit Span Backwards Test. Subjects also completed the Snellen Eye Test, which is a measure of visual acuity (Table 1).

**Table 1:** Demographic and cognitive variables between groups.

**Demographic Variables**	**Control group**	**AD group**	***t*(df)**	***X*^**2**^(*df*)**	***p***
	**(*n* = 50)**	**(*n* = 38)**			
Age (*M, SD*)	75.46 (5.254)	77.26 (6.729)	−1.41 (86)		0.177
Years education (*M, SD*)	16.05 (2.752)	15.47 (3.211)	0.91 (86)		0.378
Female (*n*, %)	32 (64%)	19 (50%)		1.737 (1)	0.188
DSF (*M, SD*)	6.12 (0.961)	5.97 (1.093)	0.67 (85)		0.516
DSB (*M, SD*)	4.50 (1.182)	4.14 (1.159)	1.43 (85)		0.154
MoCA (*M, SD*)	25.64 (2.097)	18.97 (3.578)	10.90 (85)		<0.001

*AD, Alzheimer's group; DSF, Digit Span Forward; DSB, Digit Span Backward; MoCA, Montreal Cognitive Assessment*.

### Data Analysis

Descriptive statistics were used to summarize the characteristics of the groups as well as incidence and frequency of the feasibility measures. *T*-tests, chi-square (2-sided), and Fisher's exact tests were used as appropriate to compare differences between the control and AD group with respect to the measures. All analyses were performed using SPSS version 24. The significance level was set at *p* < 0.05.

## Results

The mean age of the groups was 75 years (SD 5.25) for the control group, and 77 years (SD 6.73) for the AD group. The groups were similar with respect to education, sex, ethnicity, and socio-economic status. However, not surprisingly, groups differed with respect to cognition, with the mean Montreal Cognitive assessment score of 25.64 (SD 3.0) for the control group and 18.61 (SD 3.67) for the AD group; *t* = 10.85 (61), *p* < 0.0001, indicating cognitive impairment in the AD group.

### VR Feasibility

**Figure 2:**
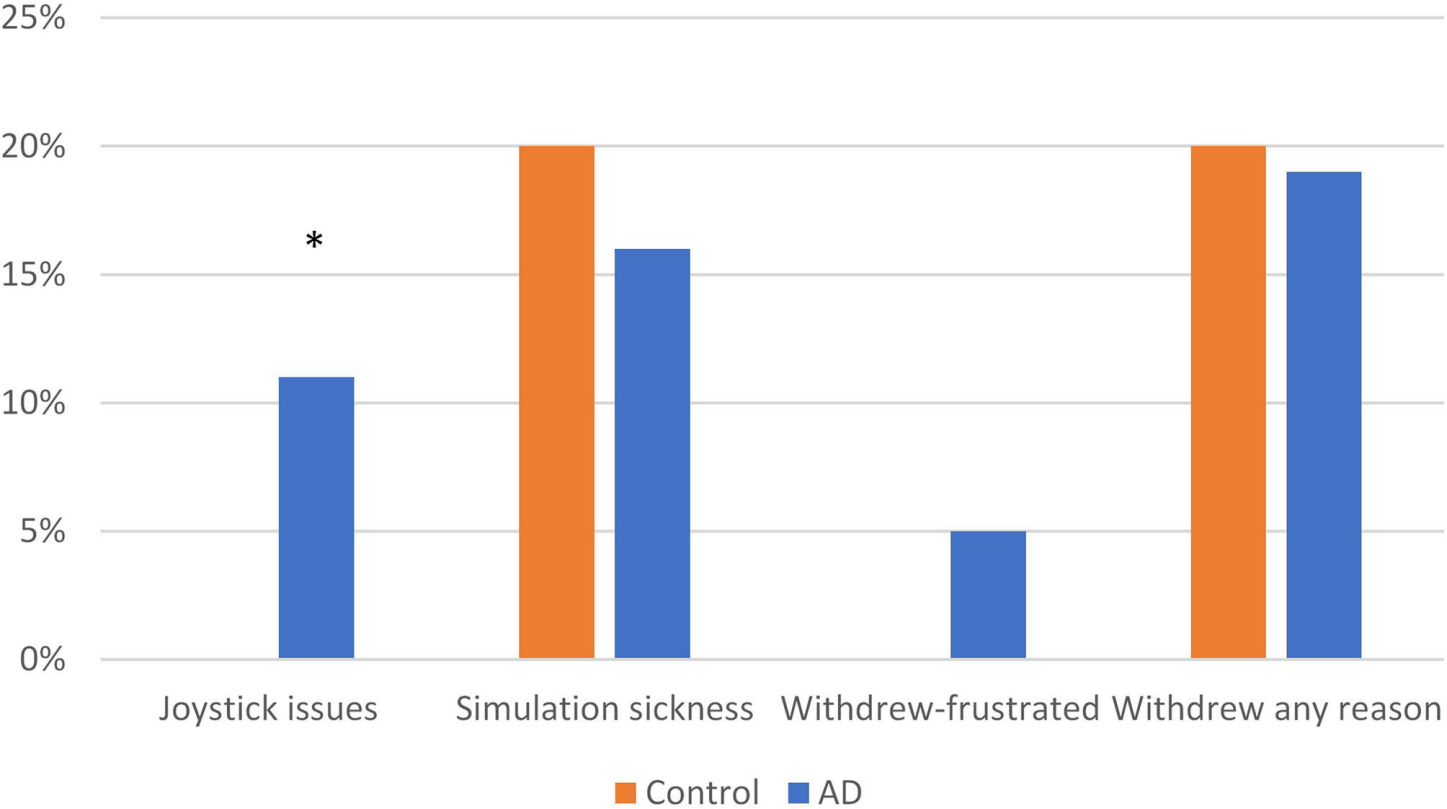
Feasibility measures: virtual reality. ^*^p < 0.05.

The VR feasibility measures are displayed in Figure 2. For the entire sample, the majority (91%) of the subjects demonstrated ability to use the joystick, with only 4 subjects (9%) who could not, rendering them ineligible to participate in the study. All four subjects who could not use the joystick were in the AD group (11% of the group; Fisher's exact test, *p* = 0.041). Of who met the inclusion criteria (*n* = 88), 70 (80%) subjects were able to complete all of the VR testing consisting of 10 trials for each of 2 consecutive days. The ability to complete the VR testing was not significantly different between the groups; (Control, *n* = 40, 80%; AD, *n* = 30, 79%); *X*^2^_(1)_ = 0.015, *p* = 0.903. Of those participants who withdrew, 18% (*n* = 16) withdrew due to simulation sickness symptoms (nausea, lightheadedness) and 2 (2%) withdrew due to frustration with the VR task. Of the 16 who withdrew due to simulation sickness, 14 withdrew on the first day of testing, ranging from 2 to 5 trials into the study (on average after the fourth trial). The AD and Control groups were not significantly different with respect to simulation sickness [*X*^2^_(1)_ = 0.257, *N* = 88, *p* = 0.612] or frustration (Fisher's Exact test; *p* = 0.184). The two subjects who withdrew due to frustration were in the AD group.

### Eye Tracking Feasibility

**Figure 3:**
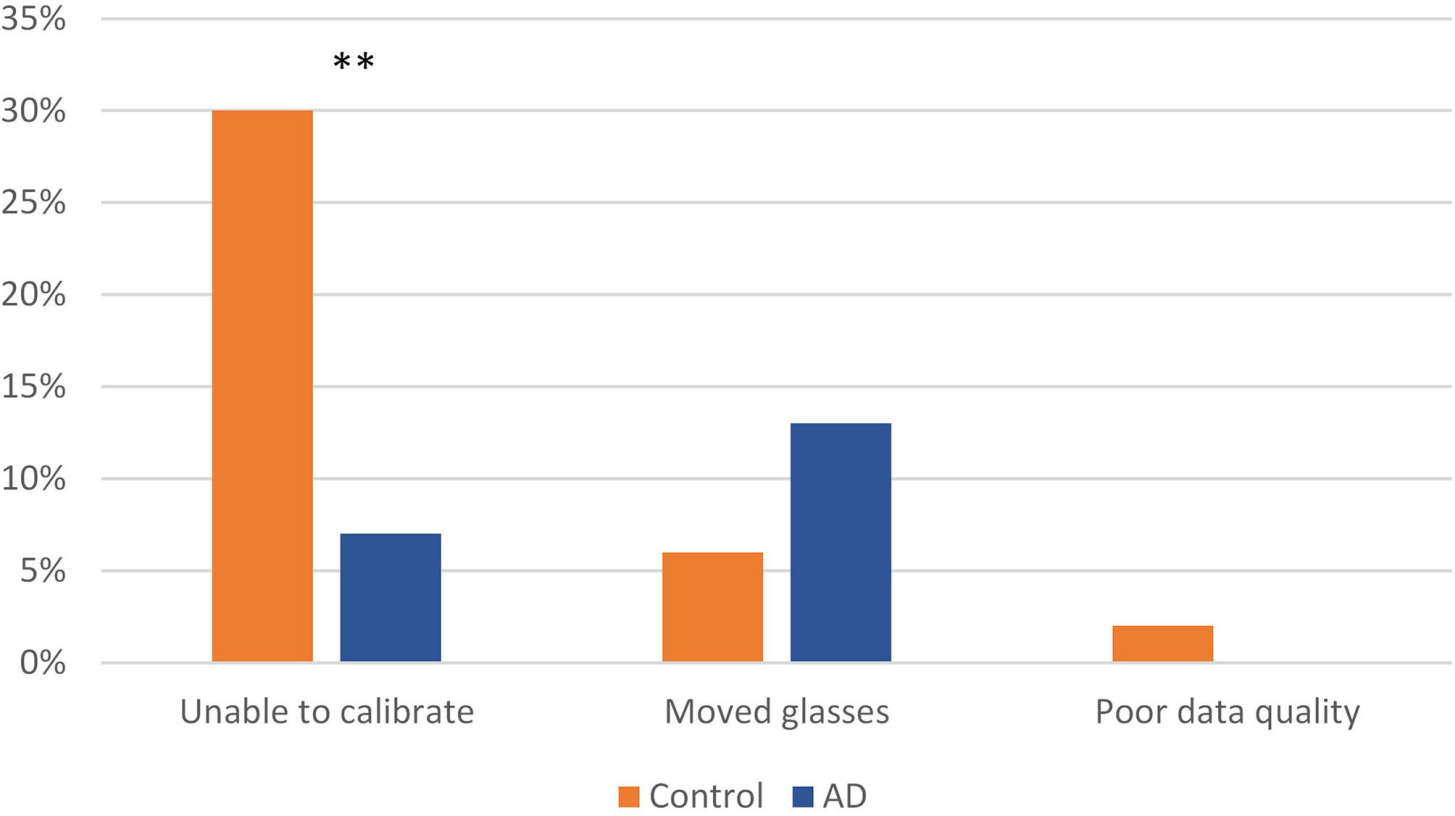
Feasibility measures: eye tracking. ^**^p < 0.01.

Eye tracking feasibility measures are depicted in Figure 3. Of those participants who completed all 20 trials over the 2 days of study (*N* = 70), 42 (60%) had complete, usable eye tracking videos. Sixteen (23%) were unable to be eye tracked, and 12 (17%) had partially usable eye tracking videos. The main reason for inability to eye track was difficulty in calibration; many subjects had trifocal glasses with lines separating the lenses; and others had irregular pupils or cataracts. Significantly more subjects in the AD group were able to be calibrated (*N* = 35/38, 92%) than the control group (*N* = 34/50, 68%); *X*^2^_(1)_ = 7.411, *N* = 88, *p* = 0.006. A total of 9 (9%) subjects, 3 (8%) in the control group and 6 (13%) in the AD group, had incomplete eye tracking videos due to moving their eye tracking glasses (thus losing calibration during testing) with no differences between the groups (Fisher's Exact Test; *p* = 0.294).

## Discussion

The results of this study provide evidence that projected VR and eye tracking can be used in older adults with and without AD in a large-scale wayfinding task, but that there are some limitations. For the VR, there was a relatively moderate incidence of simulation sickness. This rate of simulation sickness was not significantly different between the groups, indicating that simulation sickness did not occur more or less frequently in persons with AD than the control group. However, more subjects with AD had difficulty moving the joystick than those without AD. Cognitively, understanding how to move a joystick toward a location may be difficult for persons with AD. Older adults may also have motor problems that make moving a joystick difficult. In this study, those with AD were in the early stage of the disease; as the disease progresses, movement using a joystick may be even more difficult.

The findings of participants experiencing moderate levels of simulation sickness are in alignment with other studies (Duzmańska et al., 2018). However, findings are mixed, with some studies showing that older adults do not have more simulation sickness than younger adults (Saredakis et al., 2020). The disparity in findings related to simulation sickness may be due to different types and the quality of the VR that is being used in studies. In this study, we used a projected VR on a very large (12 foot) screen because it was more immersive than a laptop screen. This type of VR was chosen because it is more immersive and realistic than a laptop screen. We did not choose to use a head mounted display, as these devices are heavier and less familiar to older adults. However, one meta-analysis of VR sickness in head mounted displays showed that overall, persons tolerated this type of VR quite well; yet they found few studies that included older adults (the mean age in the reported studies was <30 years) and none with AD(Saredakis et al., 2020). Another factor that may have increased the risk for simulation sickness in the current study was that subjects were immersed in the VR for a relatively long time—up to 30 min per day. All but two of the subjects who complained of simulation sickness had their symptoms on the first day of testing, on average after 4 trials (up to 12 min of exposure). Studies have shown that simulation sickness is related to length of exposure in younger persons (Moss et al., 2011; Sakhare et al., 2019); the current study findings would indicate that length of time of exposure is also true for older adults.

Eye tracking was challenging in this population, with almost 20% of the whole sample not able to be eye tracked, mainly due to an inability to calibrate the eye tracker. Data collectors reported that the most difficult persons to eye track were those who wore trifocal glasses with visible lines (thereby obscuring the camera) or those who had prior eye surgery with irregularly shaped pupils. These issues are common in older adults and may impact eye tracking when using the type of eye tracker that requires calibration. Obtaining a good and reliable calibration is an essential step in receiving reliable data from the eye tracker (Nyström et al., 2013). Manufacturers list wearing glasses and previous eye surgery as sometimes causing difficulty in calibration (Tobii Eye Tracking Company, 2020). It was interesting that the AD group was more likely to be calibrated than the control group; we found nothing like this reported in the literature. Visual acuity was similar between the groups and we found no objective reason for this finding; however, we did not collect data on who was wearing glasses and who was not, which is a limitation in this study. It is possible that persons with AD were less likely to be wearing glasses; but this requires further investigation. Finally, several of our subjects “lost” calibration (and thereby losing data) during trials due to moving the eye tracking glasses—due to our experimental procedure, we could not stop the study and recalibrate every time this happened. As mentioned earlier, this occurred in a total of 12 subjects, and occurred more often in the AD group, but this finding was not significantly different between the groups. It is possible that the longer exposure time wearing the glasses lead to more movement of the glasses. This resulted in some unusable data.

### Recommendations

Based on the study findings, we have the following recommendations. VR is an excellent, ecologically appropriate way to test way-finding abilities in older adults with and without AD. However, further studies should be conducted that address the type of VR that promotes the least amount of simulation sickness while maintaining participants' ability to self-navigate. Further studies on the type of eye tracking devices that are tolerated the best by older adults who wear multifocal glasses or who have had eye diseases are needed. Researchers that use mobile eye trackers with optics that require calibration (a common method of eye tracking) should expect that not all older adult subjects will be able to be eye tracked.

In addition, based on the results of this study we concluded that VR is a feasible platform for the assessment of spatial abilities in older adults with and without AD. The majority of the subjects were able to perform the task asked of them with a small number of subjects unable to use a joystick. We recommend limiting the time of exposure to the VR at any one setting. In the current study, the majority of subjects who had motion sickness experienced it an average of 12 min into the study. The current study examined wayfinding over time to simulate real world learning of places, vs. one time only learning. This required exposure of multiple trials over 2 days of testing, which increased the amount of time of exposure to the VR for each day of testing (up to 30 min each day). It is likely that spacing out the trials over more days (having less than four trials per day) would help to reduce simulation sickness. Shorter duration of exposure, then, would expected to yield fewer withdrawals due to motion sickness.

The eye tracking portion of this study yielded important results about the differences in fixations between older adults with and without AD. The eye tracking glasses used in this study are commonly used for eye tracking in more naturalistic environments, because they allow for head and body motion and movement through real space if desired. Eye tracking during life-like VR or in real environments allows for more ecologically valid information about eye movements and visual attention than eye tracking during exposure to static objects or fixed, identical routes on a computer screen. Other methods of eye tracking, such as screen-based trackers, may require head fixation and immobilization. Despite the inability to calibrate all of the subjects, we report that all subject tolerated the glasses well and did not report them as uncomfortable or distracting. In fact, no subjects withdrew from the study because of discomfort or irritation with the glasses. A major benefit was that the goggles were able to be used over existing glasses. We recommend that researchers using this method should expect to oversample to take into consideration the inability to calibrate when subjects have glasses or have had eye surgery.

### Limitations

The strengths of this study were that we recorded and analyzed the ability to use VR and eye tracking in a study of older adults with and without AD. Findings from this study should be considered in light of the limitations of this research, which included the small sample size, and self-report of symptoms. In addition, there are many types of VR and eye tracking—our study specifically involved projected VR with self-navigation with a mobile eye tracker, and may not be able to be applied to other types of VR or eye tracking. Older adults with and without AD may respond differently to these types of VR or eye tracking.

## Conclusion

In conclusion, the results of this study show that the use of large scale, self-mobile, projected VR is relatively well-tolerated in older adults and those with AD. These results support the use of this technology in this population, with the caveat that some subjects may experience simulation sickness in prolonged testing. Eye tracking is possible in research studies, and tolerated well by the participants. However, eye trackers that require calibration may be difficult to calibrate (requiring over sampling to meet target samples) for older adult population.

## Data Availability Statement

The raw data supporting the conclusions of this article will be made available by the authors, without undue reservation.

## Ethics Statement

The studies involving human participants were reviewed and approved by Grand Valley State University IRB; Mercy Health Saint Mary's IRB. The patients/participants provided their written informed consent to participate in this study.

## Author Contributions

RD conducted the study, participated in the data analysis, and writing the article.

## Conflict of Interest

The author declares that the research was conducted in the absence of any commercial or financial relationships that could be construed as a potential conflict of interest.
